# Beta-Band Functional Connectivity Influences Audiovisual Integration in Older Age: An EEG Study

**DOI:** 10.3389/fnagi.2017.00239

**Published:** 2017-08-07

**Authors:** Luyao Wang, Wenhui Wang, Tianyi Yan, Jiayong Song, Weiping Yang, Bin Wang, Ritsu Go, Qiang Huang, Jinglong Wu

**Affiliations:** ^1^Intelligent Robotics Institute, School of Mechatronical Engineering, Beijing Institute of Technology Beijing, China; ^2^School of Life Science, Beijing Institute of Technology Beijing, China; ^3^The Affiliated High School of Peking University Beijing, China; ^4^Department of Psychology, Hubei University Wuhan, China; ^5^College of Computer Science and Technology, Taiyuan University of Technology Shanxi, China; ^6^International Joint Research Laboratory of Biomimetic Robots and Systems, Ministry of Education Beijing, China; ^7^Key Laboratory of Biomimetic Robots and Systems, Ministry of Education Beijing, China

**Keywords:** functional connectivity, EEG, audiovisual integration, aging, beta band

## Abstract

Audiovisual integration occurs frequently and has been shown to exhibit age-related differences via behavior experiments or time-frequency analyses. In the present study, we examined whether functional connectivity influences audiovisual integration during normal aging. Visual, auditory, and audiovisual stimuli were randomly presented peripherally; during this time, participants were asked to respond immediately to the target stimulus. Electroencephalography recordings captured visual, auditory, and audiovisual processing in 12 old (60–78 years) and 12 young (22–28 years) male adults. For non-target stimuli, we focused on alpha (8–13 Hz), beta (13–30 Hz), and gamma (30–50 Hz) bands. We applied the Phase Lag Index to study the dynamics of functional connectivity. Then, the network topology parameters, which included the clustering coefficient, path length, small-worldness global efficiency, local efficiency and degree, were calculated for each condition. For the target stimulus, a race model was used to analyze the response time. Then, a Pearson correlation was used to test the relationship between each network topology parameters and response time. The results showed that old adults activated stronger connections during audiovisual processing in the beta band. The relationship between network topology parameters and the performance of audiovisual integration was detected only in old adults. Thus, we concluded that old adults who have a higher load during audiovisual integration need more cognitive resources. Furthermore, increased beta band functional connectivity influences the performance of audiovisual integration during normal aging.

## Introduction

In daily life, our brain must constantly combine all kinds of information in one or more cues from different sensory modalities. A large body of evidence from daily life has suggested that cognitive functions decline during normal aging. This decline brings trouble during elderly life. As auditory and visual information become more important, the study of age-related differences in audiovisual integration helps us understand the aging process.

To understand the processing of audiovisual stimuli, recent Event-related potentials (ERPs) studies analyzed the time course of visual, auditory and audiovisual stimuli (Fort et al., 2002; Stekelenburg et al., 2004). Furthermore, oscillatory responses in the alpha, beta, and gamma bands have been related to sensory processing. It may be related to harmonize activation of cell assemblies. Fu et al. (2001) showed that the alpha-suppression mechanism occurs during audiovisual stimulus with the use of auditory cues in an attention experiment. Studies with EEG and magnetoencephalogram (MEG) have suggested that the gamma band is also related to the integration of information (Basar, 2013). For the beta band, several groups discussed it during different cognitive processes. The results indicated that oscillatory beta forms an important substrate of human cognition processes, such as attention, working memory and audiovisual integration. Senkowski et al. (2006) investigated beta oscillatory facilitation behavior in an ERP study during visual, auditory and audiovisual stimuli. Sakowitz et al. (2005) found increased beta responses during audiovisual stimuli in comparison to unisensory stimuli on the basis of the intersensory component. Senkowski et al. (2008) used a sensory gating paradigm, which is an integration of meaningful semantic inputs, and they reported that crossmodal effects were related to evoked beta responses.

Recent studies have described age-related audiovisual integration. Some behavioral researches have reported enhanced audiovisual integration in older adults (Laurienti et al., 2006; Peiffer et al., 2007; Mahoney et al., 2011). However, most of the studies were behavioral studies and did not focus on different oscillatory frequency bands. There are many factors that influence the audiovisual integration, such as the location of the presented experimental (Molholm et al., 2004). When the audiovisual stimuli were presented peripherally, the integration could also be elicited. In addition, age-related differences were significant. The maximal behavioral enhancement in older adults occurred more delayer and the time window was longer than in younger adults (Wu et al., 2012). ERP and EEG studies have shown the deficits in attentional control affected the audiovisual integration (Mozolic et al., 2012). Some study using arrow as cue to investigate the age-related visuospatial attention. The results shown the performance for old and young adults is similar. In addition, old adults had slower ERP components and similarly amplitude compared to young adults (Curran et al., 2001). These findings indicate that there are some changes to audiovisual integration with aging, but the underlying neuronal mechanisms are still not fully understood.

Recent research has shown that functional interactions between brain areas are crucial for effective cognitive functioning (Wen et al., 2012), a concept referred to as “functional connectivity.” Functional connections play an important role in multisensory processing. Connections not only between sensory related subcortical structures but also between cortical areas can mediate multisensory integration (Beer et al., 2011; Bishop et al., 2012; van den Brink et al., 2014). Some studies investigated the functional network affects audiovisual integration in different ways. The network could be reorganized due to long-term training (Paraskevopoulos et al., 2015). In addition, functional connectivity could be reorganized by cognitive training (Bamidis et al., 2014; Klados et al., 2016). However, the age-related differences of functional connectivity during audiovisual integration is still unknown.

We used EEG to investigate age-related audiovisual integration; its high temporal resolution makes it rather suitable for the identification of synchronization across frequency bands. The EEG signals were recorded over brain to study the functional connectivity. The PLI, a synchronization measure, reflects the extent of inter-trial phase variability for a given frequency across time. PLI is defined as a period of phase locking between two events, and it can only be estimated in a statistical sense. It removes and attenuates the synchronization that occurs at or near the zero phase difference. From this way, we could reduce the interference of signals from common sources or volume conduction, which were regarded as spurious synchronization (Stam et al., 2007; Doesburg et al., 2013).

In this study, we sought to investigate the functional connectivity in different oscillatory frequency bands during audiovisual integration. We hypothesized that functional connection could influence the audiovisual integration and there are differences between old and young adults. To address this issue, we designed three stimuli: V, A, and bimodal audiovisual (AV) stimuli, which are presented peripherally. We combined the phase synchrony of electrode interactions and graph-theoretical metrics of network topography to investigate task-dependent functional connectivity derived from EEG data. The PLI computed for each pair of sensors was used to construct graphs in various frequency bands independently.

## Materials and Methods

### Participants

Twelve old male adults (60–78 years, mean age ± SD, 68.6 ± 4.74) and 12 young male adults (22–28 years, mean age ± SD, 23.9 ± 1.73) participated in this study. To confirm their cognitive function, all of the participants did the mini-mental state exam to identify cognitive function. Participant who had a score more than 2.5 SD from the mean score that matched his age and level of education were excluded (Bravo and Hebert, 1997). In addition, participants were excluded if they self-reported any disease. Due to the experiment requirement, all of the participants had normal or corrected-to-normal vision (none of the participants were color blind) and normal hearing capabilities. The individuals provided written informed consent, which was previously approved by the ethics committee of Okayama University.

### Stimuli and Task

The experiment was performed in a dimly lit, sound-attenuated, electrically shielded room (laboratory room; Okayama University, Japan). Stimuli presentation and response collection were determined using the Presentation software (Neurobehavioral Systems Inc., Albany, CA, United States). A 21-inch computer monitor with a black background was positioned 60 cm in the front of the participant’s eyes and was used to present visual stimuli. The auditory stimuli were presented through an earphone. Each block consisted of 300 visual stimuli, 300 auditory stimuli and 300 audiovisual stimuli. All of the stimuli were randomly presented and had an equal probability of appearing to the left or right of the central fixation point.

**FIGURE 1:**
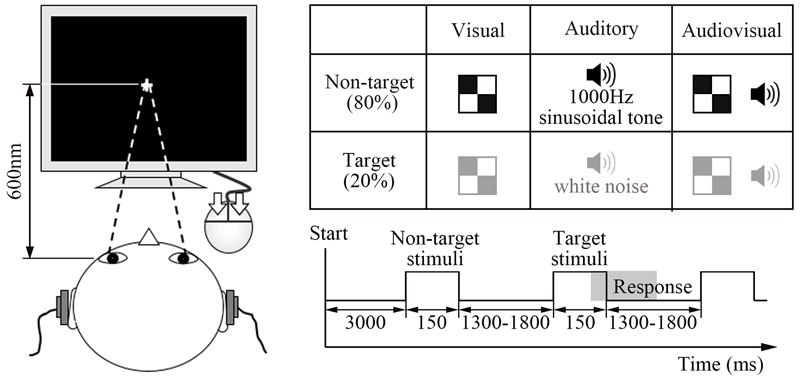
Experimental design. The visual target stimulus was a red and white block, and the non-target visual stimulus was a black and white block. The auditory target stimulus was a white noise, and the non-target auditory stimulus was a 1000 Hz sinusoidal noise. The audiovisual stimulus consisted of the simultaneous presentation of both visual and auditory target or non-target stimuli. In addition, non-target stimuli were presented at a frequency of 80% of the total stimuli.

The visual target stimulus was a red and white block, and the non-target visual stimulus was a black and white block (5.2 cm × 5.2 cm with a subtending visual angle of ∼5°). These visual stimuli were peripherally presented at an angle of ∼12° from a centrally presented fixation point in the lower visual fields (∼5° below the horizontal meridian) (He et al., 1996; Talsma and Woldorff, 2005). The auditory target stimulus was white noise, and the non-target auditory stimulus was a 1000 Hz sinusoidal tone (60 dB sound pressure level, 5 ms rise or fall time). The audiovisual stimulus consisted of the simultaneous presentation of both the visual and auditory target or non-target stimuli. In addition, non-target stimuli were presented at a frequency of 80% of the total stimuli (**Figure 1**).

At the beginning, each participant was required to complete five experimental blocks, and each block lasted ∼5 min. In formal experiment, each block had a 3000 ms fixation period, followed by the test stimulus. Each type of stimulus displayed 150 ms and continued with 1300 – 1800 ms interstimulus interval time. Within the interval time, participant responded to the target stimulus and the screen was cleared. The experiment continued regardless of whether the participant responded. Participants were instructed to click the left or right button with the forefinger or middle finger quickly and accurately when the target stimuli occurred. They were also instructed to stare at the central white cross during the whole experiment.

### EEG Data Collection

The EEG signals were recorded from 30 scalp electrodes mounted on an electrode cap (Easy cap, Germany), as specified by the International 10–20 System, and 2 electrooculogram electrodes that were referenced to the earlobes. Data were bandpass filtered from 0.05 to 100 Hz during the recordings and were digitized at a sampling rate of 500 Hz by BrainAmp amplifiers (BrainProducts, Munich, Germany).

## Data Analysis

### Interregional Phase Synchronization

Our data were pre-processed with Matlab R2013a (Mathworks Inc., Natick, MA, United States) with the following open source toolboxes: EEGLAB^1^ (Swartz Center for Computational Neuroscience, La Jolla, CA, United States). An Independent Component Analysis was used to remove artifacts (e.g., eye artifacts, muscle artifacts and electrocardiographic activity) from the data within all channels. We also corrected the baseline for each epoch.

The non-target stimuli were filtered into alpha (8–13 Hz), beta (13–30 Hz), and gamma (30–50 Hz) frequency ranges. The network synchronization of all three bands was investigated. For each subject, the Hilbert transform was employed to obtain the time series of instantaneous phase measures for each trial, source and frequency band. Phase locking was calculated for each EEG sensor pair and frequency with the PLI (Stam et al., 2007).

(1) $$PLI=\left|\langle \mathrm{sign}\left(\Delta \;\phi \left(t_{n}\right)\right)\rangle \right|=\left|\frac{1}{M}\sum_{k\;=\;1}^{M}\mathrm{sigh}\left(\Delta \;\phi \left(t_{n}\right)\right)\right|$$

At a given time point, it measures the reliability of phase relations between two EEG sensors, which produces a sensor-by-sensor adjacency matrix. Epochs were extracted from 300 ms before stimulus onset until 500 ms after stimulus onset. We removed the first and last 150 ms (75 sample points) due to the distortions caused by Hilbert transform at the edges of the epochs (Doesburg et al., 2008). These were then averaged within each group (old adults and young adults) for each trial condition (auditory, visual and audiovisual, each condition includes two orientations). The average PLI values across EEG sensors for each time point reflect task-dependent dynamic network connectivity. For each participants, we calculated it at each frequency, respectively. An two-sample *t*-test (sample size of bootstrap is 1000) was performed at each time point to compare the differential PLI value of the old and young adults. Time points with significant age differences were used to identify windows for further analyses.

### Statistical Analysis of Network Dynamics

According to the results of the two-sample *t*-test above and previous studies, adjacency matrices for non-overlapping 150 ms time-windows after stimulus onset were extracted for each frequency: 0–150 ms for alpha and gamma bands and 50–200 ms for the beta band. To characterize task-dependent network connectivity dynamics, these adjacency matrices were averaged and represent the mean connectivity within this active window for each subject.

Task-dependent network synchronization was analyzed by Network Based Statistic (NBS), which is a data-driven approach. Statistical significance of group differences could be displayed, which was corrected for multiple comparisons. (Zalesky et al., 2010, 2012). In this study, the purpose of the NBS is to identify any connected structures that are significantly different between old adults and young adults. At first, we applied a univariate statistical threshold to each element in the compared adjacency matrix, and multiple comparisons was achieved regardless of this threshold. In this case, a *t*-test was performed in the 30 × 30 adjacency matrix (*p* < 0.05) (Zalesky et al., 2010, 2012). Then, data surrogation was repeated 5,000 times to establish statistical confidence.

### Graph of Theoretical Analysis of Dynamic Network Topologies

Functional connectivity among sensors was measured by computing the PLI for every possible pair during the time window. The resulting non-linear correlation matrices were converted to weighted graphs. To characterize the task-dependent weighted network dynamics, we constructed network G (30 × 30) for each trial, frequency and subject by GRETNA (Wang et al., 2015) by using the time window identified in the above analysis.

For the constructed brain networks, we calculated brain network parameters (including the clustering coefficient, path length, small-worldness, global efficiency, local efficiency and degree) to examine both the global and regional topological characteristic variations. Each attribute was compared with those of 100 random networks. We applied a sparsity threshold (0 < *S* < 1), which normalized the networks, to examine the relative network organization. In addition, task-dependent areas under the curve (AUCs) of parameters were calculated for each network measure to provide a scalar that did not depend on the specific threshold selection.

### Statistical Analysis

SPSS version 20.0 (SPSS, Inc., Chicago, IL, United States) was used for statistical analyses. For each frequency, repeated-measures ANOVAs were carried out separately for the averaged adjacency matrices of PLI and task-dependent AUC of eight network parameters. A 2 (age group: old, young) × 3 (sensory modality: A, V, or AV) × 2 (stimuli direction: left, right) repeated-measures ANOVA analysis was performed separately to examine the effects of audiovisual integration and age as well as their interaction. The Greenhouse-Geisser epsilon value was obtained in all cases in which the repeated-measures data failed the sphericity test (Greenhouse and Geisser, 1982). All statistical comparisons were two-tailed with α = 0.05. We used the Bonferroni correction to correct for the effect of multiple comparisons in neural oscillations.

### Relationship between Network Topology Parameters and Behavior Data

Trials with target stimuli were extracted for behavior analysis. A race model was used to identify whether audiovisual integration occurred (Miller, 1986). For each participant, the target stimuli were analyzed with CDFs for the V, A and AV stimuli. In addition, the CDFs for the V, A and AVstimuli were generated using 10 ms time bins. At each time bin, the distribution of race model was calculated by the following formula: [P (V) + P (A)] – [P (V) × P (A)]. Each participant’s race model curve was then subtracted from their AV CDF. The peak time point of each probability difference curve was recorded, which represented the response time at that the audiovisual integration most likely occurred. A one-way ANOVA was performed to compare age differences (two-tailed with α = 0.05, Bonferroni correction).

Furthermore, at each oscillatory frequency, a Pearson correlation was conducted to test the relationship between each network parameter and behavior peak time point.

## Results

### Time Courses of Average PLI

**FIGURE 2:**
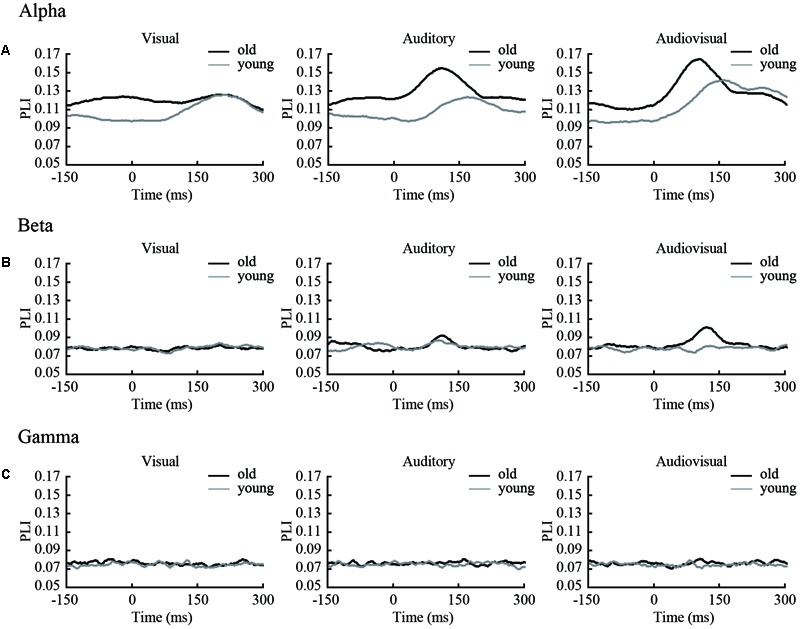
Time courses of average PLI in three frequencies. **(A)** PLI of each time point (including 150 ms before stimulus onset and 300 ms after stimulus onset) in the alpha band. **(B)** PLI of each time point (the same as in the alpha band) in the beta band. **(C)** PLI of each time point (the same as in the alpha band) in the gamma band.

We filtered the EEG data into alpha (8–13 Hz), beta (13–30 Hz), and gamma (30–50 Hz) frequency ranges. The phase synchronization of these frequencies was calculated. For each time point, we averaged the PLI of each subject within groups (**Figure 2**). For subsequent analyses, no differences were found between the left and right hemi-spaces (see Supplementary Figure 1) in the strength of PLI and network topology parameters, and we averaged the results of the two orientations.

**FIGURE 3:**
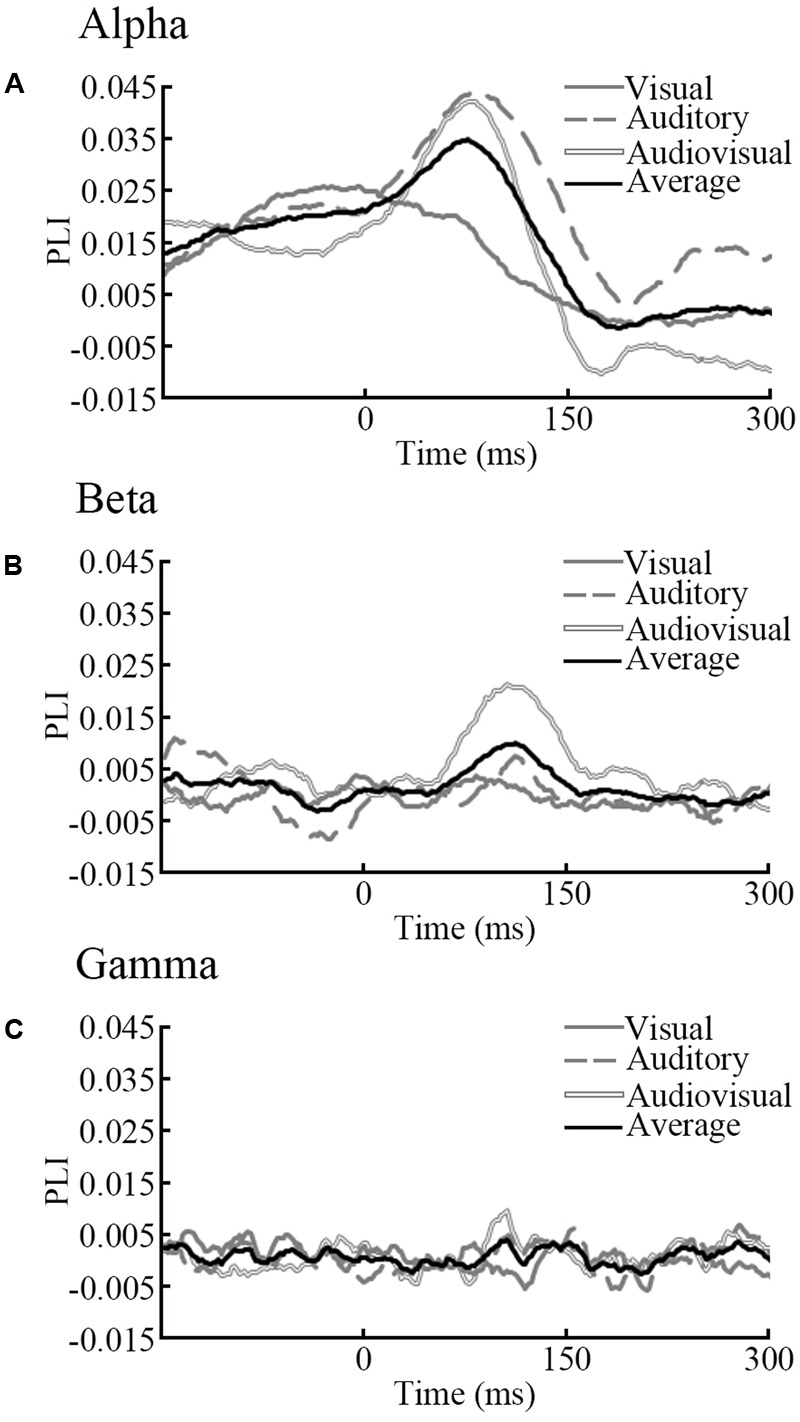
Subtraction of PLI. We subtracted the PLI of young adults from the PLI of old adults for visual, auditory and audiovisual stimuli for each frequency band. The black solid line is the average of each stimuli condition. **(A)** Results in the alpha band **(B)** Results in the beta band **(C)** Results in the gamma band.

The results show that in both the alpha and beta band, there were clear dynamic changes after stimulus onset for all condition, especially for the audiovisual stimulus. There was a significant (*p* < 0.05) difference within 50–200 ms for the beta band. This finding indicates that the strength of functional connectivity in old adults is higher than in young adults. In alpha and gamma band, only a small part of the time point within 0–150 ms was different between groups. To compare the differences between groups, we performed a subtraction for each condition. The main results obtained from our studies are summarized in **Figure 3**.

### Topographical Analysis between Groups

**FIGURE 4:**
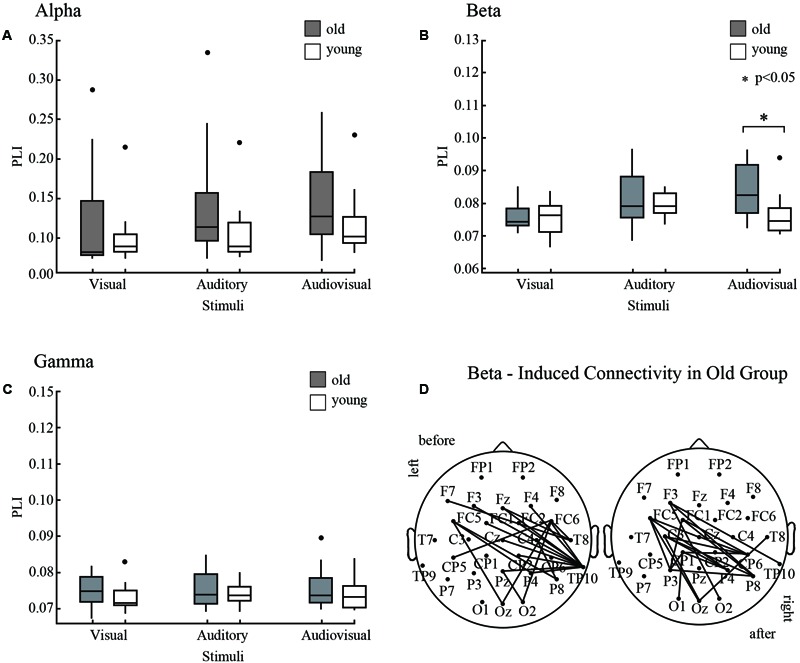
Results of NBS. We detected differences between old and young adults via NBS for each frequency band. **(A)** In the alpha band, PLI was averaged 0–150 ms after stimulus onset. **(B)** In the beta band, PLI was averaged 50–200 ms after stimulus onset. **(C)** In the gamma band, PLI was averaged 0–150 ms after stimulus onset. **(D)** In the beta band, a significant induced connection in old adults during audiovisual processing was detected.

According to time courses of average PLI and previous ERP studies (Fort et al., 2002; Stekelenburg et al., 2004), we choose the 150 ms time window after stimulus onset (0–150 ms for alpha and gamma, 50–200 ms for beta) and averaged them to characterize the task-dependent weighted network connectivity. As presented in **Figure 2**, we averaged the results of the two orientations (**Figures 4A–C**). In beta band, results of repeated-measures ANOVA shown that there is significant interaction between group and stimuli type [*p* = 0.02, *F*(2,44) = 4.800]. The simple effect results showed that there are significant differences between old and young participants during the AV target stimulus task [*p* = 0.03, *F*(1,22) = 5.39] (**Figure 4B**). The results that contains two orientations are in Supplementary Figure 2.

A strict statistical analysis was performed with Network Based Statistics to investigate group differences in phase synchrony during each condition for each frequency range. In the beta band, NBS revealed induced connectivity in the old group in a distributed network of EEG sensors only in the AV stimulus condition (*p* < 0.05, corrected, two-tailed) in **Figure 4B**. However, a left or right stimulus activated contralateral brain results in different pairwise connections of two orientations (**Figure 4D**). No significant group differences were observed for other stimulus trials.

However, NBS only reveals connected structures that are significantly different between groups. It does not reveal any statistically significant differences in topological properties.

### Statistical Results in Task-Dependent Network Topology Parameters

To assess age differences in brain network connectivity during the task, the AUC of the clustering coefficient (Cp), path length (Lp), small-worldness (Gamma, Lambda, Sigma), global efficiency (Eg), local efficiency (Eloc), and degree was analyzed. For each frequency, a 2 (age group: old, young) × 3 (sensory modality: A, V, or AV) × 2 (stimuli direction: left, right) repeated-measures ANOVA was performed.

The detailed results are shown in **Table 1**. Differential parameters between groups are presented in larger fonts. In the alpha band, there are significant interactions for the Cp value [*p* = 0.04, *F*(2,44) = 6.367]. In the beta band, there are significant interactions for Cp [*p* = 0.049, *F*(2,44) = 3.255], Gamma [*p* = 0.04, *F*(2,44) = 6.642], Sigma [*p* = 0.02, *F*(2,44) = 7.412], Lp [*p* = 0.013, *F*(2,44) = 4.820], Eg [*p* = 0.021, *F*(2,44) = 4.374], Eloc [*p* = 0035, *F*(2,44) = 3.739], and Degree [*p* = 0.014, *F*(2,44) = 4.721] values. In the gamma band, there are significant interactions for Gamma [*p* = 0.30, *F*(2,44) = 4.054], and Sigma [*p* = 0.036, *F*(2,44) = 3.781] values.

**Table 1:** Statistical results of network topology parameters for each condition.

		Visual		Auditory		Audiovisual	
		Old	Young	Old	Young	Old	Young
Alpha	Cp	0.0103 ± 0.0019	0.0119 ± 0.0024	0.0119 ± 0.0019	0.0108 ± 0.0029	0.0114 ± 0.0018	0.0115 ± 0.0020
	Lp	0.5006 ± 0.1513	0.5050 ± 0.1215	0.4196 ± 0.1305	0.4973 ± 0.1258	0.4007 ± 0.1237	0.4483 ± 0.1269
	Gamma	0.0451 ± 0.0048	0.0466 ± 0.0078	0.0449 ± 0.0057	0.0442 ± 0.0066	0.0434 ± 0.0060	0.0429 ± 0.0065
	Lambda	0.0508 ± 0.0008	0.0511 ± 0.0012	0.0510 ± 0.0007	0.0505 ± 0.0009	0.0514 ± 0.0012	0.0509 ± 0.0012
	Sigma	0.0444 ± 0.0048	0.0456 ± 0.0074	0.0439 ± 0.0054	0.0437 ± 0.0063	0.0423 ± 0.0058	0.0421 ± 0.0060
	Eg	0.0057 ± 0.0025	0.0054 ± 0.0019	0.0068 ± 0.0028	0.0055 ± 0.0021	0.0069 ± 0.0022	0.0062 ± 0.0023
	Eloc	0.0054 ± 0.0027	0.0056 ± 0.0023	0.0069 ± 0.0029	0.0055 ± 0.0026	0.0070 ± 0.0027	0.0061 ± 0.0023
	Degree	0.7425 ± 0.4244	0.6638 ± 0.2622	0.8799 ± 0.4435	0.6806 ± 0.2599	0.9258 ± 0.3597	0.7535 ± 0.2804
Beta	Cp	0.0259 ± 0.0040	0.0290 ± 0.0034	0.0284 ± 0.0040	0.0281 ± 0.0032	0.0271 ± 0.0035	0.0300 ± 0.0036
	Lp	1.2825 ± 0.1058	1.3105 ± 0.1332	1.2165 ± 0.1607	1.2419 ± 0.1071	1.1466 ± 0.1577	1.3038 ± 0.1188
	Gamma	0.0986 ± 0.0044	0.0978 ± 0.0039	0.0968 ± 0.0049	0.0975 ± 0.0039	0.0950 ± 0.0048	0.0999 ± 0.0047
	Lambda	0.1005 ± 0.0008	0.1004 ± 0.0006	0.1006 ± 0.0010	0.1008 ± 0.0007	0.1008 ± 0.0010	0.1005 ± 0.0006
	Sigma	0.0982 ± 0.0044	0.0974 ± 0.0037	0.0962 ± 0.0052	0.0968 ± 0.0038	0.0942 ± 0.0049	0.0944 ± 0.0039
	Eg	0.0078 ± 0.0007	0.0077 ± 0.0008	0.0084 ± 0.0012	0.0081 ± 0.0007	0.0089 ± 0.0015	0.0077 ± 0.0008
	Eloc	0.0080 ± 0.0007	0.0079 ± 0.0009	0.0086 ± 0.0013	0.0084 ± 0.0009	0.0092 ± 0.0017	0.0080 ± 0.0009
	Degree	0.4270 ± 0.0408	0.4200 ± 0.0434	0.4584 ± 0.0688	0.4455 ± 0.0408	0.5006 ± 0.1163	0.4231 ± 0.0479
Gamma	Cp	0.0110 ± 0.0025	0.0128 ± 0.0021	0.0118 ± 0.0019	0.0124 ± 0.0025	0.0114 ± 0.0026	0.0118 ± 0.0016
	Lp	0.7597 ± 0.0680	0.7981 ± 0.0546	0.7513 ± 0.0704	0.7921 ± 0.0475	0.7572 ± 0.0702	0.7923 ± 0.0627
	Gamma	0.0464 ± 0.0042	0.0503 ± 0.0039	0.0474 ± 0.0043	0.0485 ± 0.0043	0.0481 ± 0.0044	0.0473 ± 0.0045
	Lambda	0.0500 ± 0.0005	0.0502 ± 0.0003	0.0503 ± 0.0006	0.0501 ± 0.0003	0.0501 ± 0.0004	0.0501 ± 0.0004
	Sigma	0.0463 ± 0.0040	0.0501 ± 0.0039	0.0471 ± 0.0045	0.0483 ± 0.0043	0.0480 ± 0.0043	0.0472 ± 0.0045
	Eg	0.0033 ± 0.0003	0.0031 ± 0.0004	0.0034 ± 0.0003	0.0032 ± 0.0002	0.0033 ± 0.0003	0.0032 ± 0.0003
	Eloc	0.0030 ± 0.0005	0.0030 ± 0.0004	0.0033 ± 0.00060	0.0029 ± 0.0002	0.0032 ± 0.0006	0.0029 ± 0.0004
	Degree	0.3906 ± 0.0349	0.3742 ± 0.0291	0.3972 ± 0.0373	0.3797 ± 0.0238	0.3962 ± 0.0413	0.3787 ± 0.0305

*The size of indexes that significantly different between groups was not larger.*

The simple effect results showed that there are significant trends for old individuals to have lower Cp or Gamma (normalized value of Cp) values during the visual task than young individuals for the alpha band [*p* = 0.045, *F*(1,22) = 4.53], beta band [*p* = 0.036, *F*(1,22) = 5.01], and gamma band [*p* = 0.02, *F*(1,22) = 12.60]. In addition, for the beta band, all of the parameters showed significant differences between old and young participants during the AV target stimulus task for Cp [*p* = 0.049, *F*(1,22) = 4.35], Gamma [*p* = 0.003, *F*(1,22) = 11.57], Sigma [*p* = 0.002, *F*(1,22) = 12.49], Lp [*p* = 0.008, *F*(1,22) = 8.42], Eg [*p* = 0.018, *F*(1,22) = 6.58], Eloc [*p* = 0.024, *F*(1,22) = 5.90], and Degree [*p* = 0.024, *F*(1,22) = 5.86].

### Relation to Behavior

**FIGURE 5:**
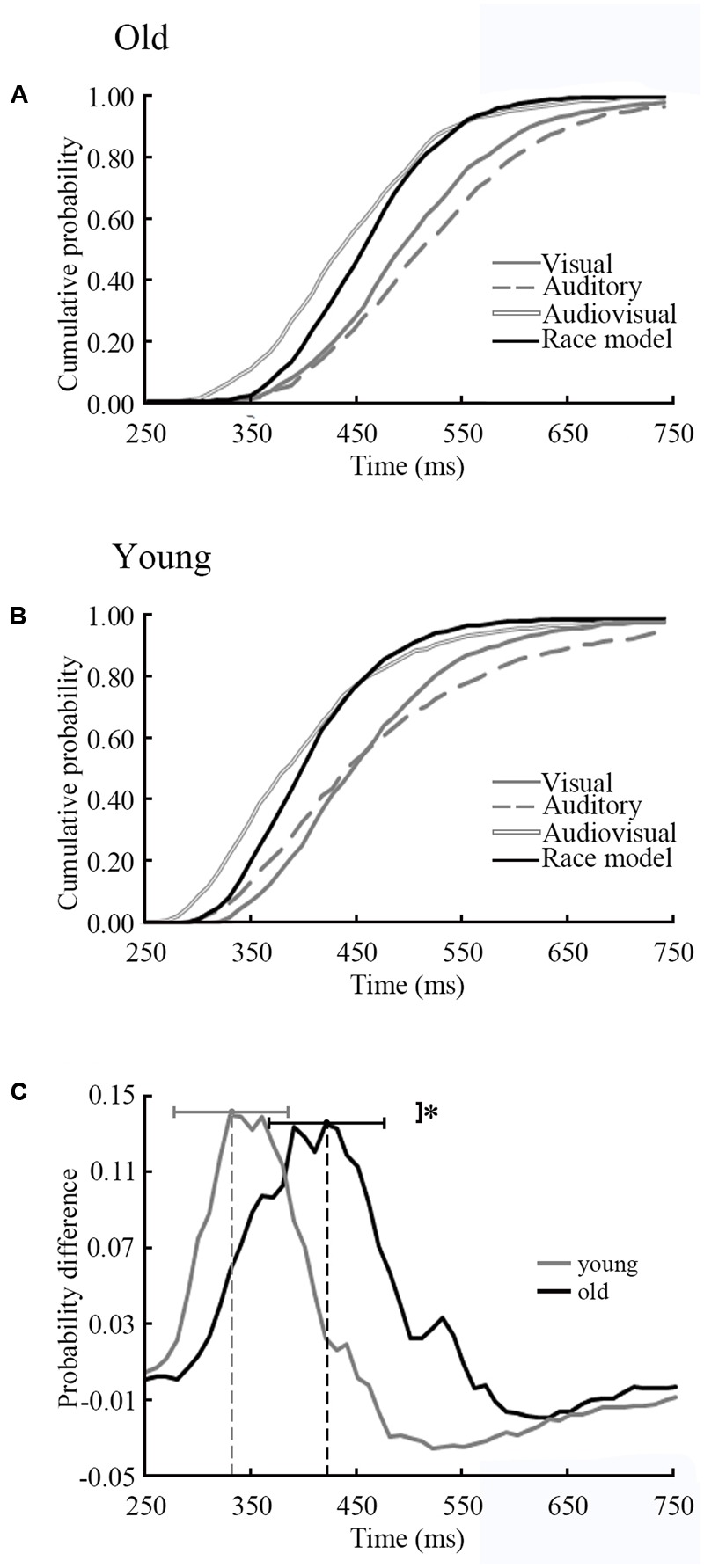
Distributions of response time of old and young adults. **(A)** CDFs for response times to visual, auditory and audiovisual stimuli in young adults. The summed probability of visual and auditory responses is shown by the race model curve (race model). **(B)** CDFs in old adults. **(C)** The cumulative probability difference curve for old adults (solid line) and the young adults (gray line). The peak time point is significant difference between two groups. ^∗^ *p* < 0.05.

The results of CDFs of V, A, AV and race model was shown in **Figure 5A** (old adults) and **Figure 5B** (young adults). The distribution of CDFs revealed that the responses to the AV stimuli were faster than the response to V or A stimuli in both age groups. Furthermore, to identify whether audiovisual integration occurred, we measured the response time to the AV stimuli by subtracting the race model for each age group independently (**Figure 5C**). The time window of behavioral facilitation in older adults was longer and more delayed than that in the younger adults. The peak time point of each probability difference curve was recorded for each participant in each age group. There was a significant difference between groups in terms of peak time point (*p* = 0.02). For the young adults, the average peak time point was 330 ms (SD: ± 56 ms), whereas the old adults had delayed response times at 420 ms (SD: ± 55 ms).

**FIGURE 6:**
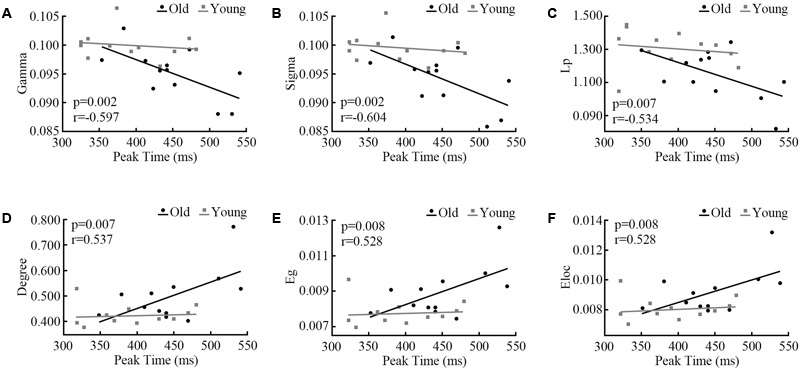
Pearson correlations between network parameters **(A)** Gamma, **(B)** Sigma, **(C)** Lp, **(D)** Degree, **(E)** Eg, **(F)** Eloc and the peak time point of cumulative probability difference curves. We only detected significant correlations in the beta band during audiovisual stimuli.

At each oscillatory frequency, a Pearson correlation was conducted to test the relationship between network parameters and behavior response. No significant relationships were observed for the alpha and gamma bands. For the beta band, only for the AV stimulus, the network topology parameters showed a significant relationship with the peak time point (**Figure 6**). It is interesting that only old adults showed a strong relationship.

## Discussion

### Summary

Neuroanatomical changes have been recognized and are thought to account for the cognitive declines during aging. However, the underlying neuronal mechanisms in age-related audiovisual integration are still unclear. This study explored this question by analyzing phase synchronization and the graph-theoretical network of EEG data. Studies have shown that EEG is rather suitable for the identification of synchronization across frequency bands in functional networks (Stam et al., 2007). Synchronous oscillatory neural activity is a possible candidate mechanism for the coordination of neural activity between functionally specialized brain regions.

The goal of the present study was to clarify the age-related functional connectivity in alpha, beta and gamma bands during visual, auditory and audiovisual stimuli. We found age differences within 200 ms after stimulus onset, which is consistent with previous studies (Fort et al., 2002; Stekelenburg et al., 2004). The results show that old adults have stronger functional connectivity while performing the same tasks, especially for audiovisual stimuli. Furthermore, beta oscillatory network connectivity influences the performance of audiovisual integration during normal aging.

### Age-Related Beta Band Functional Connectivity in Audiovisual Integration

Oscillatory phenomena corresponding to the EEG frequency bands play a major role in functional communication in the brain during cognitive process. The present study is the first to analyze age-related oscillatory functional connectivity during audiovisual integration. We focused on the alpha, beta and gamma bands, which have been related to sensory processing (Fu et al., 2001; Senkowski et al., 2006; Basar, 2013). The results indicated that age-related differences occurred during audiovisual stimuli only in the beta band. von Stein and Sarnthein (2000) showed that the beta band serves as a communication mechanism between distant cortical areas. These findings confirmed that the beta band connection plays an important role in visual, auditory and audiovisual processes during aging (Sakowitz et al., 2005; Senkowski et al., 2008).

Both old and young adults showed increased PLI in beta bands after stimulus onset. In addition, only in the beta band, old adults had a significantly higher PLI during audiovisual processing (**Figure 4B**), which indicates the presence of stronger phase locking while performing tasks. As presented in this study, we avoid effects of motor responses and analyzed only non-target stimuli. Some studies reported the same results in responses to both target and non-target stimulation (Missonnier et al., 2007; Kukleta et al., 2009). One explanation of our results is that the beta band connection increases with higher load during normal aging, which suggests that the amount of processing resources allocated to audiovisual tasks is larger for old than young adults. To achieve audiovisual tasks, old adults need to activate more beta band connection than young adults. Our result is in line with previous studies that old adults exhibit larger responses in the beta frequency range during cognitive processing (Sebastian et al., 2011; Sallard et al., 2016). Hong et al. (2016) used a Go/NoGo task to examine the effects of aging on brain networks, and showed increased phase synchrony in the beta band that was more robust in old adults. Zarahn et al. (2007) showed an increased beta response during the memory load task. In addition, researches have reported that lower cognitive reserve was related to higher functional connectivity (Lopez et al., 2014).

The NBS results shown the connected structures are significantly different between groups. Statistical results in task-dependent network topology parameters confirmed this difference. Furthermore, the peak time point of each probability difference curve was different between old and young adults (**Figure 5C**). The peak time point represents the likely occurrence of audiovisual integration. In the beta band, network topology parameters of audiovisual processing showed strong correlations with peak time points in old adults but not in young adults (**Figure 6**). However, no age differences were detected for unimodal stimuli. This finding indicates that beta band functional connectivity influences the performance of audiovisual integration during normal aging. Old adults need more cognitive resources to perform highly demanding tasks (Sakowitz et al., 2005), which leads to changes in communication within the cortical system. Diaconescu et al. (2013) revealed that the engaged additional regions during audiovisual stimuli compared to younger adults. Audiovisual integration requires a higher level of cognition than visual or auditory processing and requires an old adult to think. However, young adults do not need to try to achieve tasks that lead to a low relationship with behavior results. Our results are supported by the study by Steffener et al. (2014), who revealed the relationship between cognitive performance and functional brain activity. These previous findings suggested that increased functional brain activity relates to worse (slower) task performance in old adults but not in young adults.

Therefore, our study is in good accordance with previous studies, which showed that audiovisual integration is different between old adults and young adults. Furthermore, the oscillatory beta network functional connectivity increased and graph characteristics changed during normal aging, which influence the reaction to audiovisual stimuli.

In the future, we will determine how to adjust beta band functional connectivity to benefit audiovisual integration during normal aging. Because our participants included old adults who are unable to adapt the long-term experiment, we chose 30 scalp electrode channels to construct the brain network. One main limitation of this study may be that the node of the network is relatively small.

## Ethics Statement

This study was carried out in accordance with the recommendations of ethics committee of Okayama University with written informed consent from all subjects. All subjects gave written informed consent in accordance with the Declaration of Helsinki. The protocol was approved by the ethics committee of Okayama University.

## Author Contributions

LW analyzed and interpreted the data, wrote the paper. WW analyzed and interpreted the data. JS, WY, and QH performed the experiments. BW and RG conceived and designed the experiments. TY and JW revised the paper, approved the final version.

## Conflict of Interest Statement

The authors declare that the research was conducted in the absence of any commercial or financial relationships that could be construed as a potential conflict of interest. The reviewer EP and handling Editor declared their shared affiliation, and the handling Editor states that the process nevertheless met the standards of a fair and objective review.

## Footnotes

1 http://sccn.ucsd.edu/eeglab/

## Abbreviations

A: unimodal auditory stimulus
AUC: area under the curve of parameters
AV: bimodal audiovisual stimulus
CDFs: cumulative distribution functions
EEG: electroencephalography
PLI: phase lag index
V: unimodal visual stimulus
