# Supplementary material for: Beta-Band Functional Connectivity Influences Audiovisual Integration in Older Age: An EEG Study
Source: Front Aging Neurosci. 2017 Aug 7;9:239. doi: 10.3389/fnagi.2017.00239 (PMC5545595; doi:10.3389/fnagi.2017.00239)
Supplement: Supplementary file 1 [file Data_Sheet_1.PDF]

Supplementary Material

Beta-Band Functional Connectivity Influences Audiovisual Integration in Older Age: An EEG Study

Luyao Wang, Wenhui Wang, Tianyi Yan, Jiayong Song, Weiping Yang, Bin Wang, Ritsu Go, Qiang Huang and Jinglong Wu

*** Correspondence:** Tianyi Yan: yantianyi@bit.edu.cn; Jinglong Wu: wujl@bit.edu.cn

1 Supplementary Figures and Tables

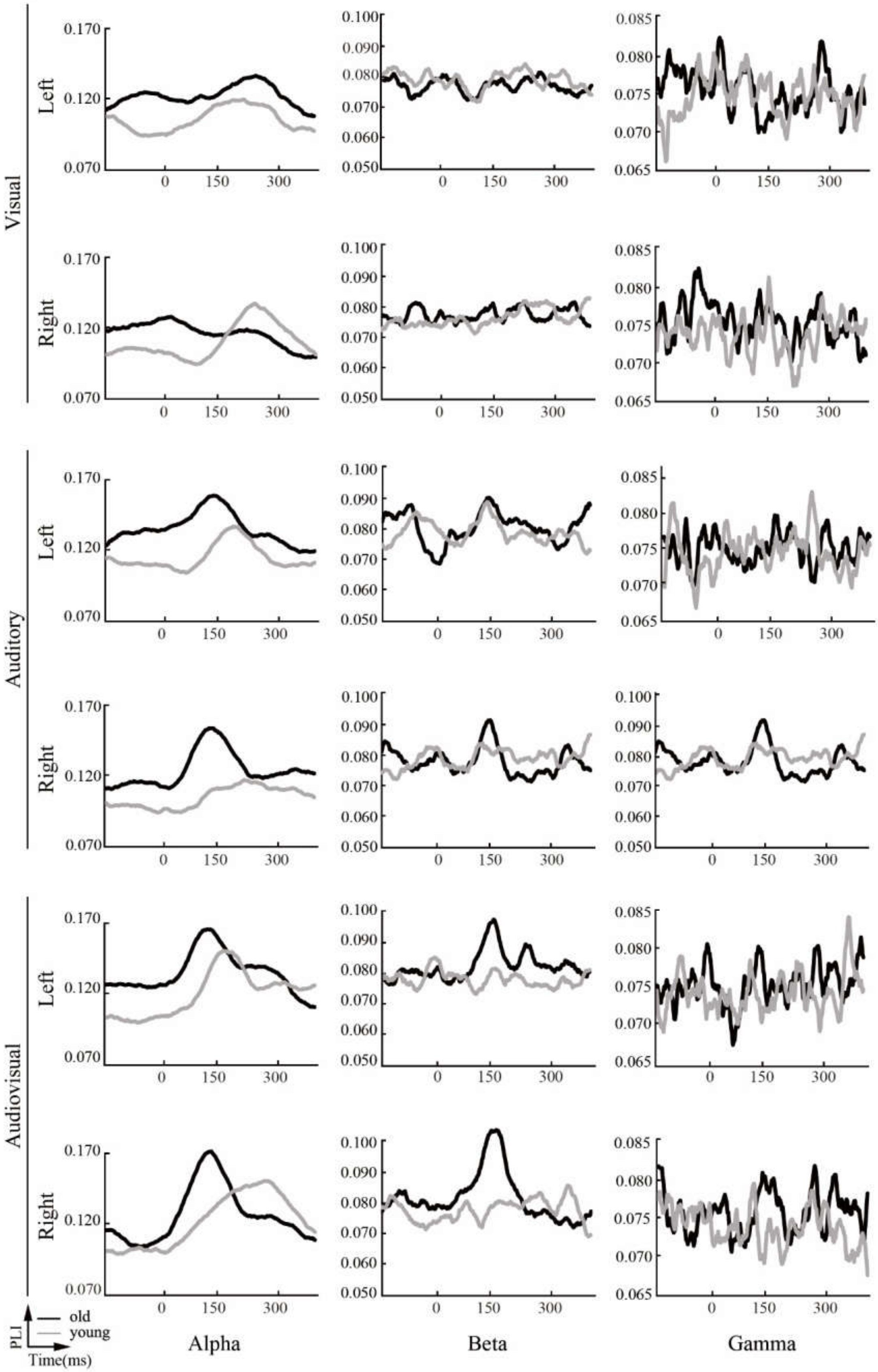

Visual
Auditory
Audiovisual
Left
Right
0.170
0.120
0.070
0.100
0.090
0.080
0.070
0.060
0.050
0.085
0.075
0.065
0
150
300
old
young
PLI
Time(ms)
Alpha
Beta
Gamma

Supplementary Figure 1. Time courses of average PLI in three frequencies that contains two orientations.

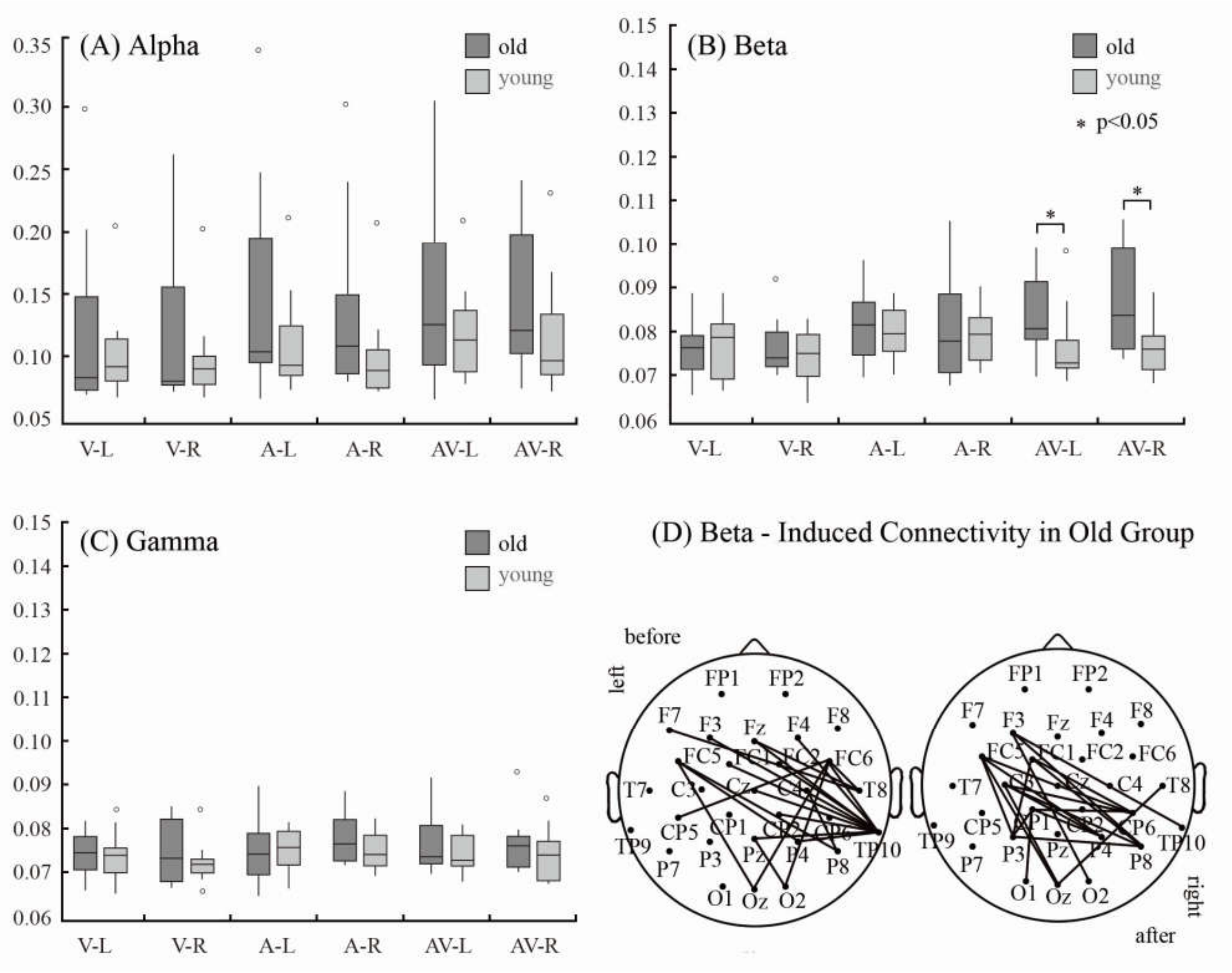

Supplementary Figure 2. Results of NBS that contains two orientations.
